# Anxiety Symptoms and Associated Factors Among Chronic Low Back Pain Patients in China: A Cross-Sectional Study

**DOI:** 10.3389/fpubh.2022.878865

**Published:** 2022-05-04

**Authors:** Yueming Hu, Zechuan Yang, Yong Li, Yong Xu, Xuan Zhou, Ningfeng Guo

**Affiliations:** ^1^Department of Orthopedics, Hwa Mei Hospital, University of Chinese Academy of Sciences, Ningbo, China; ^2^Ningbo Institute of Life and Health Industry, University of Chinese Academy of Sciences, Ningbo, China; ^3^Department of Orthopedics, Tongji Hospital, Tongji Medical College, Huazhong University of Science and Technology, Wuhan, China; ^4^Department of Anesthesiology, Tongji Hospital, Tongji Medical College, Huazhong University of Science and Technology, Wuhan, China

**Keywords:** anxiety symptoms, chronic low back pain, associated factors, prevalence, China

## Abstract

**Background:**

This study aimed to investigate the prevalence of anxiety symptoms among patients with chronic low back pain and explore its related factors.

**Methods:**

A cross-sectional study was conducted on patients with chronic low back pain from two general hospitals in China. Anxiety symptoms were assessed by the Generalized Anxiety Disorder-7. Binary logistic regression was used to examine the association between demographic characteristics, pain severity, pain self-efficacy, family functioning and anxiety symptoms.

**Results:**

This study involved 1,172 chronic low back pain patients, with an effective rate of 94.67%. The prevalence of anxiety symptoms among patients with chronic low back pain in China was 23.89%. In the binary logistic regression, patients with more severe pain (OR = 1.15, 95%CI: 1.11–1.18) and pain duration between 1~5 years (1~3 years: OR = 2.45, 95%CI: 1.38–4.36; 3~5 years: OR = 2.99, 95%CI: 1.49–6.00) had a higher risk to anxiety symptoms. In contrast, patients with higher monthly income (OR = 0.62, 95%CI: 0.39–0.98), better family functioning (highly functional family: OR = 0.22, 95% CI: 0.13–0.37; moderately dysfunctional family: OR = 0.44, 95% CI: 0.27–0.72) and higher pain self-efficacy (OR = 0.95, 95%CI: 0.94–0.96) had a lower risk to anxiety symptoms.

**Conclusion:**

The prevalence of anxiety symptoms among chronic low back pain patients was high in China. Targeted intervention measures should be taken to reduce anxiety symptoms levels of chronic low back pain patients.

## Introduction

Chronic low back pain (CLBP) is a public health problem with the disease characteristics of long duration of illness and high disability rate, which has caused widespread concern worldwide. Studies have shown that patients with CLBP often have limited physical activity due to long-term painful symptoms. Those with severe conditions may occur disabilities, reducing their quality of life (1, 2). In addition, the regular medical treatment and limited ability to work caused by CLBP can also impose a heavy financial burden on patients (3). Under the dual influence of economic and disease burdens, patients with CLBP bear a substantial psychological burden and are highly susceptible to psychological disturbance (4).

As one of the common psychological problems, anxiety symptoms have a high prevalence in patients with CLBP (5). A national study in South Korea showed that anxiety symptoms were much higher in patients with CLBP than in the general population (42.5 vs. 18.2%) (6). Patients who reported CLBP among adults aged 18–65 years in the Netherlands were also at significantly increased risk of developing anxiety symptoms (7). Some scholars speculate that for patients with CLBP, psychological factors may have a greater impact on disability and quality of life than pain itself (8). On the one hand, pain patients with comorbid anxiety symptoms tend to evaluate themselves negatively, thereby underestimating their ability to cope with difficult situations and increasing their perception of pain symptoms (9). On the other hand, combined psychological problems can also increase direct healthcare costs for patients with CLBP (10). In recent years, some countries have recognized the importance of psychological issues in the treatment of patients with CLBP (11), such as the Denmark (12) and the United States of America (13), which recommended early identification of psychosocial factors contributing to poor patient outcomes when conducting cognitive therapy. Therefore, it is necessary to explore the prevalence of anxiety symptoms among patients with CLBP.

Previous studies have shown that demographic characteristics and clinical variables are related to anxiety symptoms in CLBP patients. In terms of demographic characteristics, patients of female gender (14) and low education levels (15) were reported to be at high risk of experiencing anxiety symptoms. However, there are few data on the impact of other characteristics on anxiety symptoms in CLBP patients. Concerning the clinical variables, a Dutch study showed that pain intensity was significantly associated with anxiety symptoms in CLBP patients, but the association between pain duration and anxiety symptoms was not observed (16). A study conducted in India did not find a significant association between the severity of pain and anxiety (17). Families are the primary source of social support for CLBP patients, a protective determinant of mental health. Studies have reported in cancer (18) and hypertensive patients (19) that better family functioning is associated with lower anxiety symptoms levels, but few studies have been performed in CLBP patients.

China has a large population of patients with CLBP and the number of sufferers has been increasing in recent years, with the prevalence rate of 17.6% (20), significantly higher than Portugal (10.4%) (21), the United States (10.2%) (22), and Japan (3.9%) (23). CLBP has become one of the major diseases that contribute to the disease burden of the Chinese population, especially in terms of life years lost due to disability (24). However, faced with such a heavy disease burden, only one study conducted in China reported the levels of anxiety and depression symptoms in patients with LBP, and the results showed that patients scored higher than normal levels of anxiety and depression (25). In this study, we hypothesized that the prevalence of anxiety symptoms in CLBP patients is high in China, and demographic characteristics, clinical variables, and family functioning would be significantly associated with anxiety symptoms. The aim of this study is to determine the prevalence and related factors of anxiety symptoms among CLBP patients, so as to provide evidence for interventions to reduce the anxiety symptoms of CLBP patients.

## Materials and Methods

### Study Design and Participants

This was a cross-sectional study following the Strengthening the Reporting of Observational Studies in Epidemiology (STROBE) reporting guideline. One general hospital in Ningbo, Zhejiang, and one in Wuhan, Hubei, were selected as the study sites using the convenience sampling method. Patients with CLBP (≥3 months duration) aged 18 years and above who was native Chinese speaker and gave informed consent to participate in the study. Patients who had trauma or surgery on the low back within the past year, were pregnant within the past year, and were diagnosed with severe mental illness were excluded.

### Data Collection

Data collection was carried out from May to August 2021 by clinicians in the orthopedics department. All clinicians underwent uniform training and a pilot survey before the investigation to ensure consistency in the data collection process. During the study period, clinicians first identified potential subjects based on inclusion and exclusion criteria, and recruited subjects consecutively for the study. The clinicians distributed the questionnaire to participants after they signed informed consent. If participants were illiterate or visually impaired, clinicians read out the questions and answers to them.

### Measures

The anxiety symptom was the dependent variable assessed by the Generalized Anxiety Disorder-7 (GAD-7) scale, which consisted of seven items. Respondents answered each item using a 4-point Likert scale ranging from zero (not at all) to three (almost every day). The total score of the scale was 0–21, where a total score ≥10 indicated anxiety symptoms and < 10 indicated no anxiety symptoms (26). The GAD-7 has been widely used in the Chinese population and showed good reliability and validity (27). The Cronbach's α of this scale in this study was 0.95.

According to the previous literature, the questionnaire included demographic characteristics, such as gender, age, marital status, education level, work status, monthly personal income and medical insurance, which may be related to the anxiety symptoms.

The pain severity was assessed using the Chinese version of the Pain Severity subscale of the Brief Pain Inventory (BPI-PS) (28). This scale has been widely used in patients with LBP (29). It contained four items and asked patients to rate their pain level in the four situations: worst in the last 24 h, least in the last 24 h, average in the last 24 h, and right now. Each item was scored from 0 (no pain) to 10 (most severe pain). The total score of the pain severity scale was 0–40, and the higher the score, the more severe the pain. The Cronbach's α for this scale in the present study was 0.92.

The Family Adaptation, Partnership, Growth, Affection, Resolve (APGAR) Scale was used to assess the general family functioning (30, 31). The APGAR contained five items measuring five aspects of family functioning: adaption, partnership, growth, affection, and resolve. Each item was evaluated by a 5-point Likert scale, ranging from 0 (hardly ever) to 2 (almost always). The total score was ranged from 0 to 10 and classified as three levels: severely dysfunctional family (0–3 scores), moderately dysfunctional family (4–6 scores), and highly functional family (7–10 scores). This scale was validated in the previous studies with good reliability and validity in Chinese population (32, 33). In the present study, Cronbach's α was 0.88.

Pain self-efficacy was measured by the Chinese version of the Pain Self-efficacy Questionnaire (PSEQ), which showed good reliability and validity in CLBP patients (34, 35). PSEQ scale had 10 items to measure the individual's confidence in completing tasks while experiencing pain. Each item was assessed on a scale of 0–6, from 0 indicating no confidence at all to 6 indicating complete confidence. The total score was ranged from 0 to 60, with higher scores indicating greater pain self-efficacy. In the present study, Cronbach's α was 0.98.

### Statistical Analysis

Data analysis was conducted using SAS 9.4 in this study. Categorical variables were presented using frequencies and percentages, and continuous variables were presented using medians and quartiles. The Chi-square test and Wilcoxon rank-sum test were used to compare differences in demographic characteristics, pain-related factors, and family functioning between patients in the anxiety and non-anxiety groups for categorical and continuous variables, respectively. Binary logistic regression was used to explore the related factors of anxiety symptoms among CLBP patients. The fit of the model was tested using the Hosmer-Lemeshow goodness of fit test. The test level was α = 0.05.

## Results

A total of 1,238 patients who met the criteria were included, of which 66 patients were excluded due to omissions in completing the questionnaire. Finally, 1,172 patients were enrolled in the analyses. The effective rate of this investigation was 94.67%.

The characteristics of CLBP patients in anxiety and non-anxiety groups are shown in Table 1. More than half of CLBP patients in the anxiety group were female, while more than half of CLBP patients in the non-anxiety group were male. The median age of patients in the anxiety and the non-anxiety groups were 52 years (IQR: 41–62) and 50 years (IQR: 37–62), respectively. The majority of CLBP patients in the anxiety and non-anxiety groups were married, had the highest education in secondary school, worked as non-manual workers, had a low-income monthly income, had health insurance, had pain duration of less than 6 months, and had highly functional family. The median score of pain severity was 22 (IQR: 14–25) and 11 (IQR: 8–16), and the median score of pain self-efficacy was 17.5 (IQR: 5–39.5) and 46 (IQR: 35–53) in the group of anxiety and non-anxiety, respectively.

**Table 1:** The clinical characteristics of chronic low back pain patients in anxiety and non-anxiety groups.

	**Non-anxiety**	**Anxiety**	***P***
	**(*n* = 892)**	**(*n* = 280)**	
**Gender**			0.0061
Male	466 (52.24%)	120 (42.86%)	
Female	426 (47.76%)	160 (57.14%)	
**Age**	50 (37–62)	52 (41–62)	0.0113
**Marital status**			0.7438
Unmarried/divorce/widowed	158 (17.71%)	52 (18.57%)	
Married	734 (82.29%)	228 (81.43%)	
**Education level**			<0.0001
Primary school or below	208 (23.32%)	60 (21.43%)	
Secondary school	231 (25.90%)	117 (41.79%)	
Senior high school	136 (15.25%)	41 (14.64%)	
Junior college	144 (16.14%)	28 (10.00%)	
University or above	173 (19.39%)	34 (12.14%)	
**Work status**			0.9852
Unemployed	287 (32.17%)	90 (32.14%)	
Manual work	260 (29.15%)	83 (29.64%)	
Non-manual work	345 (38.68%)	107 (38.21%)	
**Monthly personal income**			<0.0001
<5000 RMB	487 (54.60%)	195 (69.64%)	
≥5000 RMB	405 (45.40%)	85 (30.36%)	
**Medical insurance**			<0.0001
Yes	820 (91.93%)	225 (80.36%)	
No	72 (8.07%)	55 (19.64%)	
**Duration of pain**			<0.0001
<6 months	281 (31.50%)	133 (47.50%)	
6 months to 1 years	250 (28.03%)	38 (13.57%)	
1 to 3 years	214 (23.99%)	49 (17.50%)	
3 to 5 years	72 (8.07%)	33 (11.79%)	
>5 years	75 (8.41%)	27 (9.64%)	
**Pain severity**	11 (8–16)	22 (14–25)	<0.0001
**Family functioning**			<0.0001
Highly functional family	542 (60.76%)	101 (36.07%)	
Moderately dysfunctional family	281 (31.50%)	96 (34.29%)	
Severely dysfunctional family	69 (7.74%)	83 (29.64%)	
**Pain self-efficacy**	46 (35–53)	17.5 (5–39.5)	<0.0001

In this study, the prevalence of anxiety symptoms in patients with CLBP was 23.89%. According to the univariable analysis, there was a significant difference between the patients in the anxiety and non-anxiety groups in gender, age, education level, monthly personal income, medical insurance, duration of pain, pain severity, family functioning, and pain self-efficacy (Table 1).

The logistic regression analysis results are shown in Table 2. Monthly personal income, duration of pain, pain severity, family functioning and pain self-efficacy were significantly associated with anxiety symptoms in patients with CLBP. Patients with monthly personal income≥5000 RMB (OR=0.62, 95%CI: 0.39–0.98), better family functioning (highly functional family: OR = 0.22, 95% CI: 0.13–0.37; moderately dysfunctional family: OR = 0.44, 95% CI: 0.27–0.72) and higher pain self-efficacy (OR = 0.95, 95%CI: 0.94–0.96) were less likely to suffer from anxiety symptoms. In contrast, patients with higher pain severity (OR = 1.15, 95%CI: 1.11–1.18), pain duration between 1 and 3 years (OR = 2.45, 95%CI: 1.38–4.36) and 3 to 5 years (OR = 2.99, 95%CI: 1.49–6.00) were at higher risk of anxiety symptoms.

**Table 2:** Binary logistic analysis of anxiety symptoms related factors in patients with chronic low back pain.

	**OR (95%CI)**	***P***
**Age**	1.00 (0.98–1.02)	0.7183
**Gender (Ref** **=** **‘male')**		
Female	1.22 (0.84–1.77)	0.3007
**Marital status (Ref** **=** **‘Unmarried/divorce/widowed')**		
Married	1.16 (0.70–1.92)	0.5725
**Education level (Ref** **=** **‘Primary school or below')**		
Secondary school	1.69 (0.98–2.91)	0.0587
Senior high school	1.06 (0.53–2.13)	0.8673
Junior college	1.05 (0.44–2.49)	0.9204
University or above	1.05 (0.41–2.64)	0.9263
**Work status (Ref** **=** **‘Unemployed')**		
Manual work	1.52 (0.91–2.53)	0.1119
Non-manual work	1.76 (0.96–3.21)	0.0661
**Monthly personal income (Ref** **=** **‘ <5000 RMB')**		
≥5000 RMB	0.62 (0.39–0.98)	0.0420^*^
**Medical insurance (Ref** **=** **‘Yes')**		
No	1.08 (0.62–1.89)	0.7881
**Duration of pain (Ref** **=** **‘ <6 months')**		
6 months to 1 years	0.85 (0.47–1.53)	0.5902
1 to 3 years	2.45 (1.38–4.36)	0.0022^*^
3 to 5 years	2.99 (1.49–6.00)	0.0021^*^
>5 years	1.82 (0.91–3.62)	0.0893
**Pain severity**	1.15 (1.11–1.18)	<0.0001^**^
**Family functioning (Ref** **=** **‘severely dysfunctional family')**		
Highly functional family	0.22 (0.13–0.37)	<0.0001^**^
Moderately dysfunctional family	0.44 (0.27–0.72)	0.0012^*^
**Pain self-efficacy**	0.95 (0.94–0.96)	<0.0001^**^

* *P value < 0.05,*

** *P value < 0.001, Hosmer and Lemeshow goodness of fit test (P value = 0.6282)*.

## Discussion

This study found that the prevalence of anxiety symptoms in Chinese patients with CLBP was 23.89%, which was higher than that of the general population using the same measurement tool at 10.4% (36). Since the prevalence of anxiety symptoms in the general population was investigated during the COVID-19 epidemic and is higher than the prevalence before the epidemic (36), it is suggested that patients with CLBP are at high risk of developing anxiety symptoms. This conclusion is consistent with the results of a study in South Korea (6), and the reasons may be due to the pain of the disease itself and its series of negative effects on working life. Moreover, a study in Qatar used the same measurement tool for anxiety symptoms as this study showed that anxiety symptoms in LBP patients were 10.5% (37), much lower than the prevalence in the present study. This suggests that the anxiety symptoms in CLBP patients in China are serious, and medical professionals should pay close attention to the issue.

Pain intensity is a risk factor for anxiety symptoms in patients with CLBP, which was in line with the findings in Hong Kong (25). It has been suggested that pain relief is the primary need of chronic pain patients (38). Combined with the negative correlation between pain intensity and anxiety symptoms, pain intensity should be assessed and intervened to address patient's physical and psychological aspects. In addition, the duration of pain was a correlating factor of anxiety symptoms in this study. The longer the duration of pain affects patients' daily work or activities, the more significant the decline in patients' physical and mental health, and the greater the risk of anxiety symptoms. However, no significant association was found between pain duration and anxiety symptoms after more than five years of pain duration. If patients suffer from pain is long enough, they would develop tolerance to the pain state both physically and psychologically and therefore do not significantly alter their psychological status (39).

In the present study, high income, better family functioning, higher levels of pain self-efficacy were protective factors for the presence of anxiety symptoms in patients with CLBP. Possible reasons may be as follow: first, according to the conservation of resources theory, resource loss can have a profound negative impact on individuals (40). In this study, income and family functioning are energy resources, and pain self-efficacy is an individual characteristic resource. When patients with CLBP experience the loss of these resources during their illness, they are more likely to experience stress reactions characterized by anxiety symptoms (41). Second, high incomes allow patients to afford high-quality medical services, which helps them recovery from illness and thus reduces the risk of patients developing anxiety symptoms as a result of their illness. Good family functioning could provide patients with an adequate sense of security in daily life and reduce negative emotions. When patients are under high stress, it can also reduce their psychological stress reactions like anxiety symptoms (42). Pain self-efficacy is the patient's ability to perceive control of pain and pain-related symptoms in the presence of pain (34). A meta-analysis showed that pain self-efficacy was a protective factor for patients with persistent pain (43). Patients with high levels of pain self-efficacy would be more confident in pain self-management and seek pain relief treatment, thus contributing to disease recovery and maintaining a healthy state of mind (44). Therefore, it is necessary for medical professionals to provide pain education to patients with CLBP to enhance their competence and confidence in pain management, thereby reducing the risk of anxiety symptoms.

To our knowledge, this is the largest survey to investigate the prevalence of anxiety symptoms and its associated factors in Chinese patients with CLBP. This study also has some limitations. First, the study used a cross-sectional design, and it is difficult to determine the causal associations between the independent variables and anxiety symptoms. Second, the information collected in this study was based on patients' self-report, especially the anxiety symptoms, which may be subject to recall bias. The anxiety symptoms of the patients could be determined by the physician's diagnosis in future studies.

## Conclusion

It is common for Chinese patients with CLBP to suffer from anxiety symptoms. Clinicians should pay more attention to the anxiety symptoms of CLBP patients with low income, long duration of pain, high pain severity, poor family functioning, and low pain self-efficacy. This study implied that the intervention strategies to reduce the risk of developing anxiety symptoms in patients with CLBP should target patients and their family members. Regarding patients, it would be helpful to provide them with information via self-management education. This intervention can teach patients the knowledge, skill, confidence, and communication skills needed for self-management and help them address problems like anxiety symptoms. Relieving the patient's pain through pharmacotherapy, massage therapy, and spinal manipulation could also help to reduce their risk of anxiety symptoms. Regarding family members, interventions such as providing education sessions to improve their knowledge and skills in the healthcare process applied to the patients would be helpful.

## Data Availability Statement

The datasets generated during and/or analysed during the current study are available from the corresponding author on reasonable request.

## Ethical Statement

The study was approved by the Ethics Committee of Hwa Mei Hospital, University of Chinese Academy of Sciences, Ningbo, Zhejiang, China (YJ-NBEY-KY-2021-146-01). Participants volunteered to participate in this study and all signed an informed consent form prior to the survey.

## Author Contributions

YH, XZ, and NG were responsible for the conception, design, and writing of the manuscript. YH, ZY, YL, and YX were responsible for the acquisition of data and literature research. YH was responsible for the analysis and interpretation of data. All authors contributed to the article and approved the submitted version.

## Conflict of Interest

The authors declare that the research was conducted in the absence of any commercial or financial relationships that could be construed as a potential conflict of interest.

## Publisher's Note

All claims expressed in this article are solely those of the authors and do not necessarily represent those of their affiliated organizations, or those of the publisher, the editors and the reviewers. Any product that may be evaluated in this article, or claim that may be made by its manufacturer, is not guaranteed or endorsed by the publisher.

## Abbreviations

CLBP: chronic low back pain
LBP: low back pain
GAD-7: Generalized Anxiety Disorder-7
BPI-PS: Brief Pain Inventory subscale on pain severity
APGAR, Adaptation, Partnership, Growth, Affection: Resolve
PSEQ: Pain Self-efficacy Questionnaire
OR: odds ratio
CI: confidence interval.
